# Transcriptome analyses of insect cells to facilitate baculovirus-insect expression

**DOI:** 10.1007/s13238-016-0260-y

**Published:** 2016-03-26

**Authors:** Kai Yu, Yang Yu, Xiaoyan Tang, Huimin Chen, Junyu Xiao, Xiao-Dong Su

**Affiliations:** Biodynamic Optical Imaging Center, School of Life Science, Peking University, Beijing, 100871 China; State Key Laboratory of Protein and Plant Gene Research, Peking University, Beijing, 100871 China; Peking-Tsinghua Center for Life Sciences, Peking University, Beijing, 100871 China

**Keywords:** High Five cell line, baculovirus-insect cell system, codon usage, glycosylation, signal peptide

## Abstract

The High Five cell line (BTI-TN-5B1-4) isolated from the cabbage looper, *Trichoplusia ni* is an insect cell line widely used for baculovirus-mediated recombinant protein expression. Despite its widespread application in industry and academic laboratories, the genomic background of this cell line remains unclear. Here we sequenced the transcriptome of High Five cells and assembled 25,234 transcripts. Codon usage analysis showed that High Five cells have a robust codon usage capacity and therefore suit for expressing proteins of both eukaryotic- and prokaryotic-origin. Genes involved in glycosylation were profiled in our study, providing guidance for engineering glycosylated proteins in the insect cells. We also predicted signal peptides for transcripts with high expression abundance in both High Five and Sf21 cell lines, and these results have important implications for optimizing the expression level of some secretory and membrane proteins.

**Electronic supplementary material:**

The online version of this article (doi:10.1007/s13238-016-0260-y) contains supplementary material, which is available to authorized users.

## INTRODUCTION

The baculovirus-insect cell expression system is one of the most popular platforms for recombinant protein expression. It is widely used for protein structure and function studies in academic laboratories, and facilitates massive protein production in industry (Kost et al. 2005). The two common cell lines in this binary system are Sf21 (IPLB-Sf21AE) from *Spodoptera frugiperda* (Vaughn et al. 1977), and High Five (BTI-TN-5B1-4) from ovarian tissues of *Trichoplusia ni* (cabbage looper) (Wickham et al.; Davis et al. 1992).

Protein expression in insect cells has several advantages such as high expression level and easy manipulation. In addition, difficult proteins especially eukaryotic proteins that need posttranslational processing usually fold better in insect cells than in the *E. coli* expression system (Brondyk 2009). However, compared to the mammalian cells, post-translational modifications are still limited in insect cells, with glycosylation as the most significant example (Jarvis 2003; Kost et al. 2005). Due to the defect in glycosylation, functions of some recombinant glycoproteins are impaired (Xu and Ng 2015). For example, the insect cells cannot produce sialylated N-linked glycans. In the past two decades, various efforts were made to import the mammalian glycosylation pathway related genes into the insect cells to engineer the required glycosylation modification (Castilho 2015). For example, Hollister first reported in 1998 that an engineered Sf9 cell line expressing the B4GALT1 gene could produce foreign glycoproteins with terminally galactosylated N-glycans (Hollister et al. 1998). It was also reported in 2001 that both Sf9 and High Five cells were engineered to produce sialylated proteins by adding the ST6GAL1 gene (Hollister and Jarvis 2001; Breitbach and Jarvis 2001). Hollister et al. (Hollister et al. 2002) later transformed a set of other genes to generate the SfSWT-1 cell line which produce biantennary, terminally α-2,6- and α-2,3- sialylated N-glycans. More work has been recently done to obtain more powerful insect cell lines.

Next generation sequencing technology is widely used recently in biological studies. Genomes and transcriptomes of different species are sequenced, which generate high-input information for genomic studies and molecular modifications. We believe similar information can also be explored to provide guidance to engineer new strains of insect cell line for expressing proteins with mammalian type of posttranslational modifications. Genome and transcriptome of Sf21 cell line have already been reported in 2014 and 2015, respectively (Kakumani et al. 2014; Kakumani et al. 2015); however, the global genomic information of the High Five cell line is still unknown.

We constructed and sequenced an mRNA library of the High Five cell line, assembled a reference transcriptome for function and expression studies. We analyzed some protein-expression-related problems by comparing our High Five transcriptome with the reported Sf21 transcriptome (Kakumani et al. 2015). In addition, we extracted codon usage information from their coding sequences and compared it with other expression systems and model species. We also annotated transcripts that may have glycosylation-related functions, and evaluated their expression abundance to generate the global view of glycogenes in High Five and Sf21 cell lines. High expression transcripts, which have predicted signal peptide sequences, were analyzed for predicting highly efficient signal peptide sequences for secretory protein expression.

## RESULTS AND DISCUSSION

### Reference transcriptome assembly

Considering the genome size of several reference-ready insects, a total 49.5 million 101 bp paired-end reads were sequenced and yield 4.95 Gb bases raw data, 48 million clean reads were kept after low quality reads were filtered. After reads trimming with Trimmomatic, we used Trinity pipeline to do the *de novo* transcriptome assembly and obtained 31,068 transcripts with an N50 value of 2,276 bp (Haas et al. 2013; Bolger et al. 2014).

In total, 39.4 Mb bases are assembled and the average transcript length is 1,269 bp. To reduce the redundancy of the assembly, cd-hit-est was used and transcript number was reduced to 25,234 under 90% sequence identity threshold. These transcripts data sets are the so-called ‘unigene’. 13,732 coding peptide sequences are predicted with TransDecoder. Detailed statistics numbers are shown in Table 1.

**Table 1:** Assembly statistics information

	Raw assembly	Duplicate removed assembly
Trinity ‘genes’	27,389	24,000
Trinity transcripts	31,068	25,234
GC content (%)	40.84	40.71
Median contig length (bp)	722	622
Average contig length (bp)	1269.6	1160.9
Total assembled bases (bp)	39,444,068	29,294,166

All clean reads have been submitted to NCBI SRA database under accession number SRP068276. Assembly version in this paper has been submitted to NCBI TSA database (GEEM01000000).

### Assembly assessment

**Figure 1:**
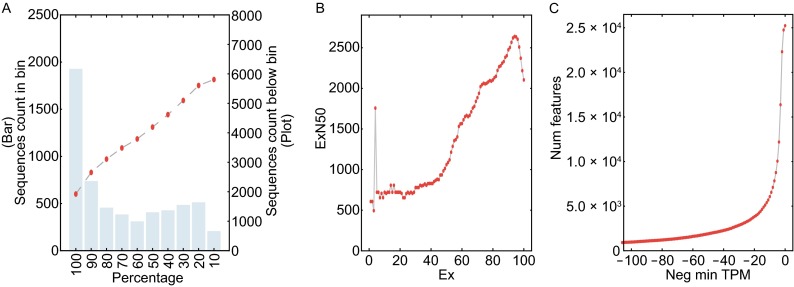
**Assembly quality assessment**. (A) Full-length transcript assessment. Bin on x-axis represent the percentage of the hit’s length included in the alignment to the Trinity transcript. Left y-axis with bar plot is the transcript count in each bin and right y-axis with point plot is the accumulate count below that bin. (B) N50 of subset of transcript by decreasing the expression level. Ex is the top most expressed transcripts that represent x% of the data. ExN50 is the length of a transcript while the total length of transcripts shorter that it reached 50% of total length of all transcripts in this dataset. (C) Transcript count with a threshold of negative minimum TPM value

To evaluate the assembly quality, we employed several strategies for quality assessment. First, we used bwa (v0.7.10) (Li and Durbin 2010) to align all clean reads back to the assembly. 97.4% of the reads could be aligned and 95.3% are properly paired, indicating that the completeness of our assembly is high and very reliable. All transcripts were then aligned to SwissProt to check the proportion of transcripts that may be full-length or near full-length. As shown in Fig. 1A, about 6000 transcripts, with more than 10% of its contig length, could be aligned to a homolog in SwissProt. Among them, about 1/3 of the transcripts are fully aligned and more than 5000 have at least 30% sequence overlapping with known homolog. The result could be underestimated as the homologs of different species’ genes have different proportion in the SwissProt database, but we believe our data reached our expectation.

Taking expression values into consideration, we recalculated the N50 value after low expression contigs were eliminated (Fig. 1B) and plotted expression value distribution pattern in Fig. 1C. Ex in Fig. 1B means a subset of top x% highly expressed transcript, the ExN50 reached the max length at E95, showing that 8,147 transcripts are in the top 95% expression subset with the minimum TPM of 6.1. From this, we can conclude from these data that most of the extremely high expression transcripts in High Five cells are in the range from 600 to 1000 bp. Longer transcripts have more regular expression level. As shown in Fig. 1C, the transcript number was reduced to 10,047 after transcripts of low TPM values (below 5) were eliminated.

### Function annotation

To annotate functions of transcripts and coding peptides, we searched homologous genes in SwissProt, TrEMBL90 and NCBI nr databases with blast. Among the 25,234 transcripts, 13,492 got blast hits in nr database, 9,639 and 13,767 have similar sequences in SwissProt and TrEMBL90 database, respectively. With 13,732 coding peptides, 9,427 and 12,198 got alignments in SwissProt and TrEMBL90 database, respectively.

**Figure 2:**
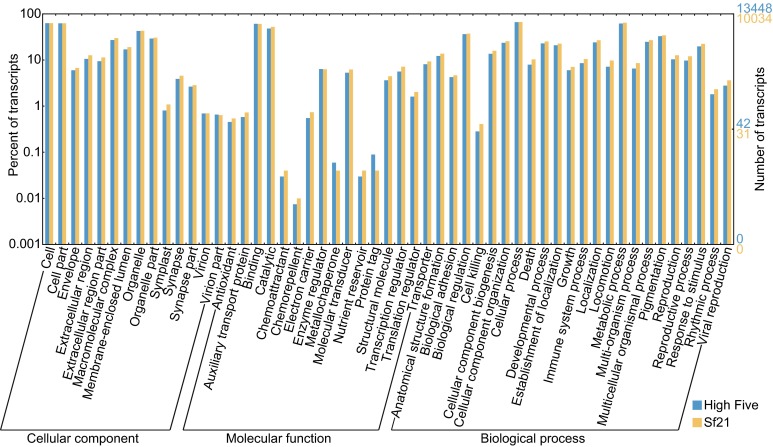
**Gene Ontology of High Five and Sf21 transcriptome**. Summarized in three main GO categories: Cellular component, Molecular function and Biological process. Right y-axis is the transcript count in that function item, left y-axis is the corresponding percentage of transcripts number

**Figure 3:**
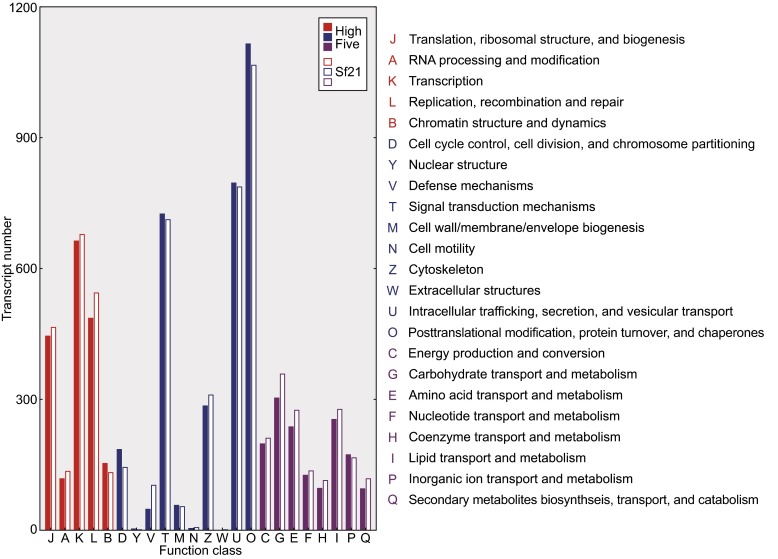
**Transcript number in each EggNOG function classes**. Divided into 3 parts by colors. (Red) Information storage and processing; (Blue) Cellular processes and signaling; (Purple) Metabolism

While executing GO annotation and EggNOG annotation, we also used protein sequence predicted from published Sf21 transcriptome data with the same analysis procedural. In High Five transcriptome, 13,447 transcripts have been annotated for GO terms and 13,459 have been annotated in EggNOG database. More specific function classification data are shown in Fig. 2 and Fig. 3, and detailed annotations for each transcript are described in supplemental file S1. From the global pattern of these figures, we can tell that the differences in GO terms and EggNOG categories between High Five and Sf21 cells are quite similar. Transcript numbers are higher for some function classes, including those related to intracellular trafficking, secretion, vesicular transport, posttranslational modification, protein turnover, transcription, translation, etc. Possessing plentiful genes with these functions make these insect cell lines an ideal host for protein production.

### Codon usage among recombinant protein expression systems

**Figure 4:**
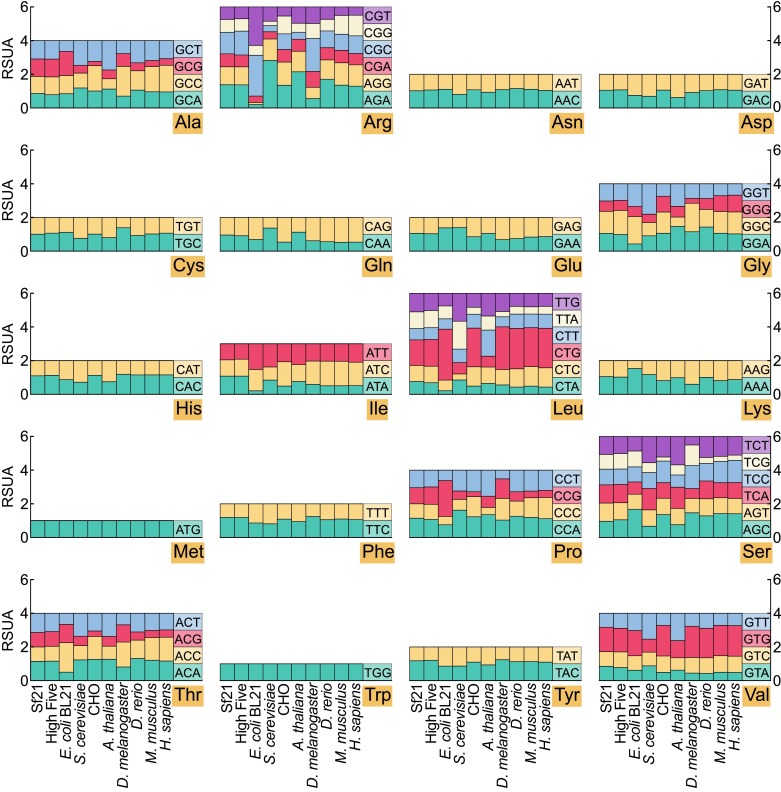
**Codon usage of 20 amino acids across 10 different species**. Each subplot is an amino acid, x-axis is different species and y-axis is the RSCU value of codons coding that amino acid

Among different organisms, synonymous codons are usually utilized with different frequencies; a phenomenon generally referred to as the ‘codon usage bias’. Codon usage bias is a major factor that affects the level of recombinant protein expression (Holm 1986). We collected CDS sequences from the Sf21 and High Five cells and compared their codon usage preference to other commonly used expression hosts, including the prokaryotic system *E. coli* BL21, the eukaryotic expression system *S. cerevisiae* and mammalian system CHO. We also compared the codon preference of insect cells with five other species including human, mouse, drosophila, zebrafish and arabidopsis. RSCU (relative synonymous codon usage) values are used to compare the use of synonymous codons. RSCU values of all amino acids in 10 species’ CDS sequence were calculated separately for downstream studies as shown in Fig. 4.

With the characteristic of RSCU value, all x synonymous codon RSCU values of an amino acid always get a sum up value equals to x. For amino acids only encoded by one or two codons, there is no extreme distribution in it, but others are quite different. We calculated the RSCU range value of a set of codon related to one amino acid. By comparing this value, we can tell in which species this set of codon have greater bias. For example, the range value of arginine, isoleucine, leucine and proline in BL21 reached 2.29, 1.32, 2.81 and 1.63, while the minimum codon’s RSCU is only 0.13, 0.21, 0.21 and 0.49. This situation exactly indicates that optimization of codon usage is of great significance. For instance, if BL21 is used to produce recombinant protein, failure to avoid these minimum codons may dramatically reduce the expression level.

In comparison with the range and standard deviation values in all species, no matter which amino acid you are using, both High Five and Sf21’s range value are at a relatively low level. The homogeneity of codon usage in baculovirus-insect system could be an advantage for protein expression. Coding sequence cloned from most species could be normally expressed in insect cells without codon optimization. This robust property of codon usage in baculovirus-insect system made it a good platform for both eukaryotic and prokaryotic recombinant protein expression. But some previous publications claim that codon with lower RSCU value is intended to slow down the translation speed in order to produce well-folded proteins (Chaney and Clark 2015). More experimental evidence is required to show whether the usage of codon with relative higher RSCU values could become a disadvantage for expression of proteins of complex folding.

### Glycogene profile in protein expression insect cells

Post-translational modification is one of the most important characteristics of baculovirus-insect expression system. But the truncated N-glycosylation pathways in insect cells limit its application on some glycoprotein expression (Jarvis 2003). Several glyco-engineering modifications have been reported in the past two decades. Some modifications require importation of glycogenes into baculovirus-insect system. Glycosylation is mediated with complicated pathways and a number of genes are involved. Without a global gene map of the insect cells, we cannot thoroughly understand glycosylation related problems. Since GGDB and CAZy databases included genes associated with glycan synthesis procedural, we used them as references to find homologs of glycogenes in our High Five transcriptome and previous Sf21 transcriptome.

**Figure 5:**
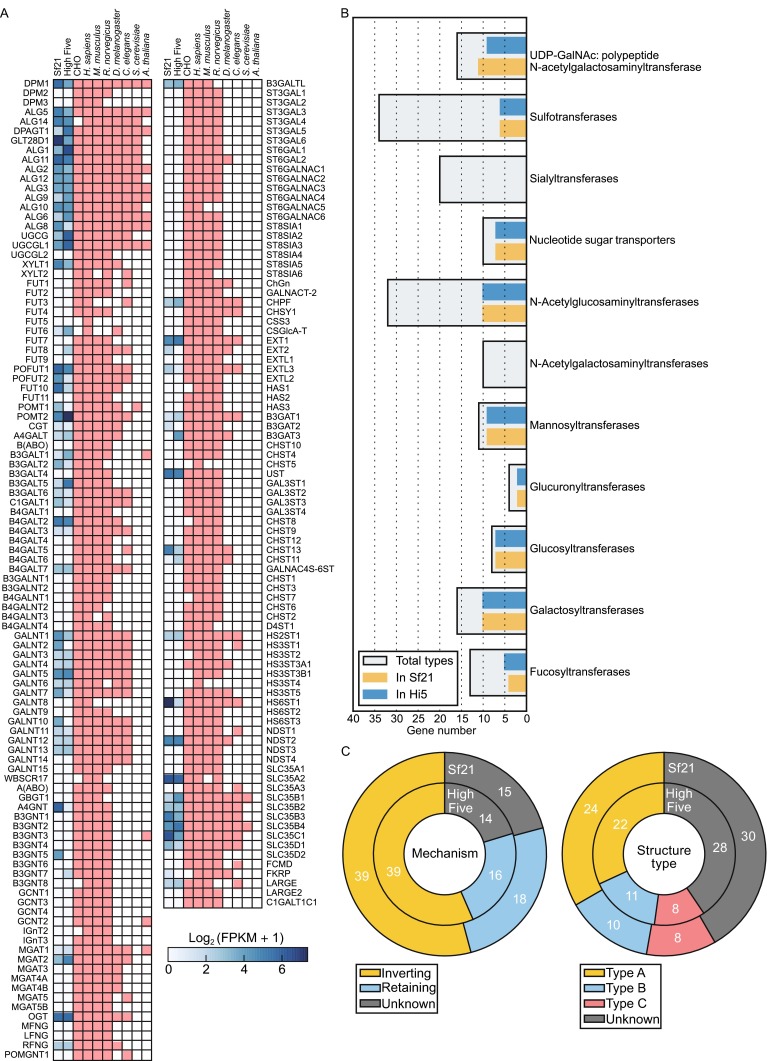
**Glycogene profile of High Five and Sf21 cell line**. (A) Heatmap represents the gene constituent of each species. Blue mark of Sf21 and High Five showed the expression value of each gene. Red mark only represent they have this gene. (B) Bar plot of glycogene categories. (C) Ring plot representing the properties of glycogenes in High Five and Sf21 cell lines

Here we identified 69 glycogenes in the High Five transcriptome and 72 in Sf21, with an overlap of 66 genes. Those genes are marked in blue with their expression value in Fig. 5A, and detailed informations are described in supplemental file S2. Glycogenes can be classified into several types according to their functions. Total gene counts of each type in High Five and Sf21 are shown in Fig. 5B. Insect cells have more or less homologs among most types. But for sialyltransferases and N-acetylgalactosaminyltransferases, no similar transcript was found in these two cell lines. That is the main reason why baculovirus-insect cannot produce complete mammalian N-glycosylation proteins. O-glycan modification is more complicated and not well studied. From the gene matrix, we found that some O-glycosylation required enzymes are detectable, such as OGT, POFUT1/2, XYLT1, POMT2, etc. Previous study suggests that O-mannosylation in insect species may occur more frequently than what is currently believed (Vandenborre et al. 2011). Understanding the profile of glycogenes in insect cells would be helpful for more detailed research on glycosylation.

We also compared the glycogenes’ functioning mechanism and structure status in High Five and Sf21 (Fig. 5C). More than 50% of the glycosyltransferases function as inverting mechanism by catalyzing group transfer with inversion at anomeric reaction center of substrate, and less than 30% are for retentions. About 30% glycosyltransferase consist of two closely abutting β/α/β Rossmann domains, 20% consist of two β/α/β Rossmann domains that face each other and is flexibly linked. Remaining part have not yet been experimentally determined or studied (Lairson et al. 2008).

Here we complemented the glycogene database with data from baculovirus-insect expression system related cell lines. Our data would be valuable for introducing supplemental mammalian glycogesnes into insect cell lines and more efficiently modifying their glycosylation properties.

### Highly expressed signal peptide containing genes

**Figure 6:**
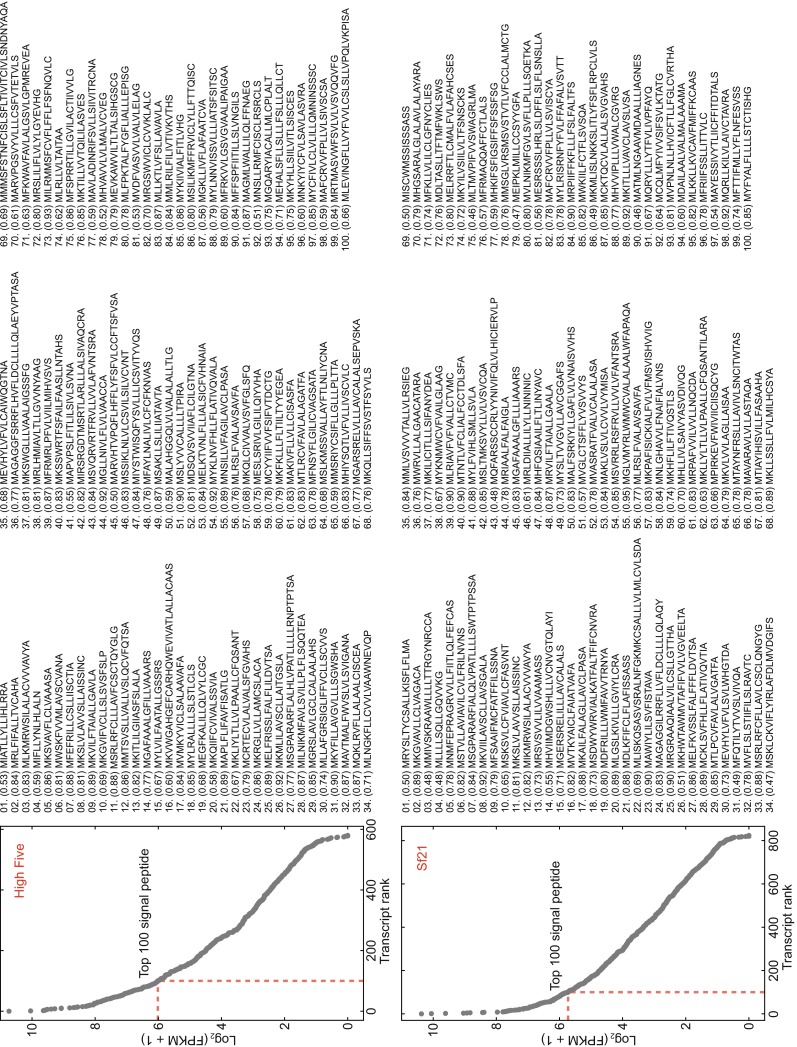
**Highly expressed predicted signal peptides**. Plot on the left are the expression values of those transcripts which have a predicted signal peptide. Sequences on the right are the top 100 signal peptide sequence with the signalP score in the brackets

Baculovirus-insect system is a good platform for secretory protein expression. When there is a signal peptide fused to the recombinant protein, product usually secreted to the outside environment through the secretory pathway. Optimization of signal peptide would be helpful to get a better yield (Olczak and Olczak 2006). Here we used SignalP software (Petersen et al. 2011) to predict all possible signal peptide in all protein sequences, and then sorted them with their transcript expression value. Fig. 6 showed all predicted transcripts that may have signal peptide sequence in High Five and Sf21 transcriptome. We identified signal peptide sequences from top 100 expressed transcripts and believe they are good candidates for higher protein production. Related peptide sequence, CDS sequence and functional annotation are described in supplementary file S3. Because higher expression value usually means higher protein amount, secretion efficiency could be closely linked with the amount of the signal peptide containing protein. Moreover, signal peptide sequence from insect itself is of the best choice because endogenous secretory signal peptide is more efficient than exogenous signal peptides (von Heijne and Abrahmsén 1989; Soejima et al. 2013). Combined with expression value and corresponding function of these proteins, we believe this approach would be useful and simplify the complexity of finding good signal peptides for protein expression.

## MATERIALS AND METHODS

### RNA extraction and library construction

High Five cell line in this study was purchased from Life technologies, USA. Cells were cultured in serum free medium containing 0.5% Penicillin-Streptomycin (Gibco 15140-122) for 24 h at 27°C in suspension at a shaking speed of 110 rpm. When cell density reached 2.4 × 10^6^/mL, 2 × 10^7^ cells were collected by centrifugation. Total RNA was extracted with QIAGEN RNeasy Mini Kit (QIAGEN, 74104) immediately after cell collection. RNA quality was examined using Agilent 2100 RNA chip (standard setting). Poly-A tailed RNA was enriched and used to construct sequencing library with Illumina TruSeq RNA Sample Prep Kit (Illumina, RS-122-2001), following standard instruction. RNA-seq library was sequenced on Illumina HiSeq 2000 platform.

### Assembly and statistics

After we got the raw reads, quality control was performed to remove poor-quality reads with in-house QC script. Then we used Trimmomatic (v0.32) (Bolger et al. 2014) software to trim low quality ends of all reads. Reference transcriptome was assembled with trimmed reads using Trinity (v2.0.6) (Grabherr et al. 2011; Haas et al. 2013). To reduce the assembly redundancy, cd-hit-est (v4.6.1) (Fu et al. 2012) was used to cluster reads with 90% identity. At this time, the remaining contigs are the so called ‘unigenes’. Coding peptide sequences were predicted with TransDecoder.

We used the SwissProt database to check the integrity of transcripts to evaluate the quality of our assembly result. All transcript sequences were aligned to SwissProt using blastx (Camacho et al. 2009), and only the most similar target was kept with the e-value cutoff of 1 × 10^−20^. Length coverage of aligned transcript was examined (Fig. 1A).

### Function annotation

Function annotation was performed at both transcript and protein level. All transcripts were aligned to SwissProt, TrEMBL90 and NCBI nr database with blastx (Camacho et al. 2009; UniProt Consortium 2015). Predicted protein sequences were aligned to SwissProt, TrEMBL90 and EggNOG 4.1 with blastp (Powell et al. 2014). We also aligned protein sequences to Pfam28 database with hmmsearch (Finn et al. 2014). Subsequently, we annotated Gene Ontology (GO) and related pathways with blast2go and KOBAS (Conesa and Götz 2008; Xie et al. 2011). The GO annotation is presented in figure with WEGO (Ye et al. 2006). We also used SignalP (v4.1) (Petersen et al. 2011) for signal peptide prediction. Since some protein sequences are 5′ truncated or predicted starting from an upstream point, we manually checked the prediction of top expression of transcripts containing signal peptides.

To compare the constituent of glycogenes, we collected glycogene sequences from GGDB as reference (Narimatsu 2004). Published Sf21 transcriptome was downloaded from TSA database with accession No. GCTM00000000 (Kakumani et al. 2015). Protein sequences were aligned to this reference with blastp. Blast results of glycogenes were manually checked by aligning sequences to the TrEMBL90 and nr databases, to eliminate false positive hits. Classification by function, mechanism and structure were based on information from GGDB and CAZy database (Narimatsu 2004; Lombard et al. 2014).

### Codon usage evaluation

We used our predicted coding sequences in codon usage evaluation. CDS sequences from other species were downloaded from Ensembl and NCBI genome databases. Only those sequences longer than 100 amino acids (300 bp CDS sequence) were used for calculation. All codons were counted with our script by shifting a simulated reading frame from 5′ end to 3′ end. With the count data of all codons, we calculated RSCU value according to the formula as described (Cannarozzi and Schneider 2012).

### Expression analysis

To evaluate the transcript abundance, Bowtie (v1.0.0) and RSEM (v1.2.15) were used for sequence alignment and calculation of TPM (Transcripts Per Million) and FPKM (Fragments Per Kilobase of transcript per Million mapped reads) (Langmead et al. 2009; Li and Dewey 2011). This value could measure the transcripts abundance with transcript length, thus we can compare it among different genes or samples.

## Electronic supplementary material

Below is the link to the electronic supplementary material.

Supplementary material 1 (XLSX 6359 kb)

Supplementary material 2 (XLSX 38 kb)

Supplementary material 3 (XLSX 35 kb)
